# Integrated nutritional care in long-term care: From theory to evidence-based practice

**DOI:** 10.1371/journal.pone.0323596

**Published:** 2025-05-30

**Authors:** Sonja Lindner-Rabl, Gerhard Wirnsberger, Barbara Bäck, Brigitte Walzl, Karola Putz-Scheicher, Johanna Fabian, Iris Wunder, Elisabeth Derler, Carolin Herzog, Lisa Pieter, Julia Traub, Lutz Kubitschke, Regina Roller-Wirnsberger

**Affiliations:** 1 Research Unit Services for Old Age and Lifelong Health, Department of Internal Medicine, Medical University of Graz, Graz, Austria; 2 Division of Nephrology, Department of Internal Medicine, Medical University of Graz, Graz, Austria; 3 Department of Medicine and Care Management, Styrian Hospital Association KAGes, University Hospital Graz, Graz, Austria; 4 Styrian Hospital Association KAGes, University Hospital Graz, Care Directorate, Graz, Austria; 5 Department of Clinical Medical Nutrition, Styrian Hospital Association KAGes, Landeskrankenhaus Hochsteiermark, Leoben, Austria; 6 Department of Clinical Medical Nutrition, Styrian Hospital Association KAGes, Landeskrankenhaus Hochsteiermark, Mürzzuschlag, Austria; 7 Department of Clinical Medical Nutrition, Styrian Hospital Association KAGes, Landeskrankenhaus Südsteiermark, Bad Radkersburg, Austria; 8 Department of Clinical Medical Nutrition, Styrian Hospital Association KAGes, University Hospital Graz, Graz, Austria; 9 Empirica Gesellschaft für Kommunikations- und Technologieforschung mbH, Bonn, Germany; South Australian Health and Medical Research Institute Limited, AUSTRALIA

## Abstract

Against the background of ageing societies and worldwide growing populations with complex care needs, the reorganization of health and social care provision from fragmented towards integrated healthcare is becoming essential. Nutritional challenges, such as malnutrition, are one of the key areas of interest, when redesigning care systems for ageing societies, especially when older people are residing in long-term care. In response to that, this study describes the development of an evidence-based inter-professional nutritional care pathway in long-term care settings. A qualitative multi-method approach of four consecutive sub-processes was performed guided by principles derived from implementation science: 1) Regional maturity assessment, 2) Matching process with existing care pathways, 3) Step-based co-creation with stakeholders, 4) Local implementation, accompanied by a planned multi-level evaluation procedure. The research process resulted in a final nutritional care pathway, structured into seven care episodes leveraging capacity around nutritional care needs of long-term care residents in an inter-professional and person-centered manner. Early evaluation results indicate that the care pathway serves as evidence-based guideline and facilitates resource-efficient practice routines in long-term care. This paper describes an iterative approach to transform nutritional care provision in long-term care. Although the concept was contextualized and developed around local needs and circumstances, it may serve as case example for subsequent integrated care efforts. Further evaluation results are needed to disclose long-term outcomes and effectiveness.

## 1. Introduction

Aging processes, chronic non-communicable diseases (NCDs) and multimorbidity [1] as well as inequality in access to health care systems [2] predispose older citizens for loss of functionality, self-care capacity and therefore care dependency. This raises the demand for care services in general and long-term care in particular, especially in middle- and high-income countries [3]. Still, quality assurance within these care structures is highly inhomogeneous between and within countries. One key quality indicator in delivery of care in resident setting is access to personalized food care [4,5], as tailored nutrition plays a key role for health for older citizens residing in resident homes and long term care [6,7]. However, the level of integration of standardized nutrition care from screening to assessment to intervention and monitoring is extremely diverse across EU countries, regions and even within different care providers on local level [8–10]. Although clinical guidelines and quality standards provide guidance and instructions for evidence-based nutritional care of older adults [11,12], there is only limited implementation of integrated approaches for person-centered and inter-professional nutritional care of long-term care (LTC) residents.

This is confirmed by recent data reporting a prevalence of high malnutrition risk of 17.5% in residential care/nursing homes [13,14], and 28.7% in long-term care [14], respectively, depending on screening tool used. As a result, residents in long-term care facilities are considerably exposed to severe health outcomes, such as functional decline, prolonged hospital stays, increased readmissions and (in-hospital) mortality [15–17].

Against this background, implementing integrated care processes and services has turned out to be challenging [18]. Although implementation science is in general clearly defined as “the scientific study of methods to promote the systemic uptake of research findings and other evidence-based practices into routine practice” [19], implementation strategies are usually characterized by a concomitant complexity, ultimately leading to challenges in terms of their precise description, operational definition and measurement options [20]. In order to overcome these challenges and provide structured guidance for implementation processes, several frameworks and guidelines have been developed [20–22], raising the potential to successfully integrate the new strategies into real-world processes, enhance effectiveness and promote the practical uptake of integrated care [18,19,23,24].

It was therefore the aim of this paper to explore the development process of an evidence-based, contextualized and inter-professional nutritional care pathway for three nursing homes of the Styrian hospital trust (KAGes Ges.m.b.H.) across the province of Styria, Austria, grounded in the Expert Recommendations for Implementing Change (ERIC) taxonomy [22].

## 2. Materials and methods

**Fig 1:**
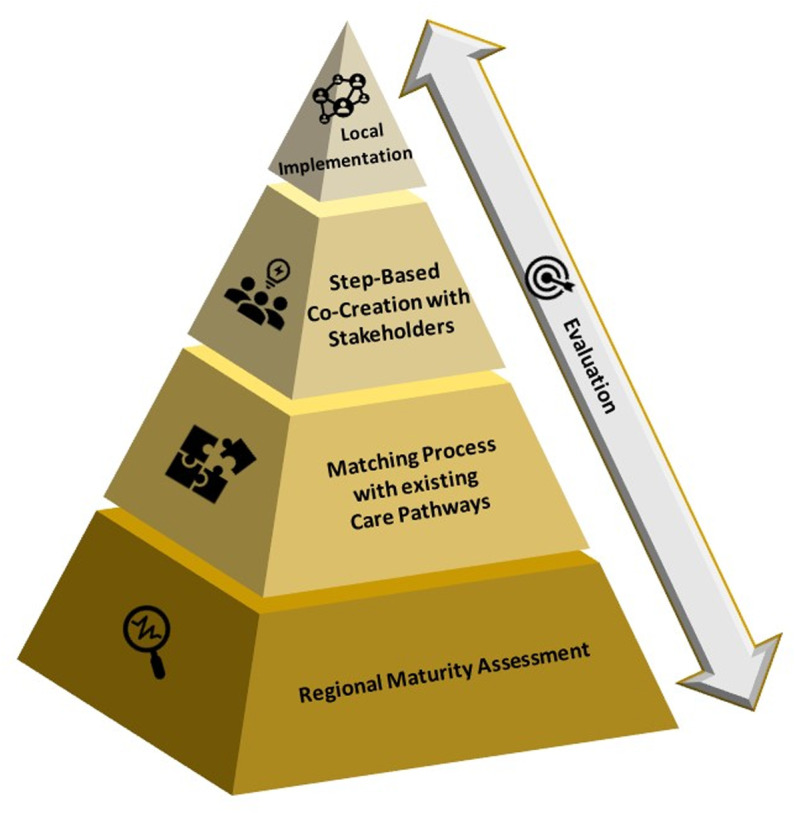
Schematic representation of the multi-method research approach. Fig 1 illustrates the multi-method research approach applied.

This publication was conducted in line with the Revised Standards for Quality Improvement Reporting Excellence (SQUIRE 2.0) publication guidelines [25] and the checklist is available as Supporting information file (S3 Checklist). Nutrition care pathway development was oriented towards the framework of the European Pathway Association for developing quality integrated care pathways [26] and based on a qualitative multi—method approach consisting of sequential and constitutive sub-processes as outlined in Fig 1.

Selected strategies from the Expert Recommendations for Implementing Change (ERIC) taxonomy [22] have been operationalized and contextually adapted. The following implementation strategies have been determined to methodologically support and guide the presented process: 1) Assess for readiness and identify barriers and facilitators; 2) Capture and share local knowledge; 3) Conduct local consensus discussions; 4) Involve executive boards; 5) Facilitation; 6) Promote adaptability.

### 2.1. Sampling

Participants were chosen with the goal of involving local experts providing pertinent information and understanding about respective in-house residential care processes, their needs and challenges. In the sense of a well-balanced representation of interests and expert fields of included professional groups, stakeholder participation is therefore justified upon their professional involvement in day-to-day (nutritional) residential care, as they bring together different perspectives, ideas and viewpoints. The participant recruitment period ran from October 11^th^, 2021 until October, 25^th^, 2021 and was primarily coordinated by the in-house dietician of each location as part of the project core group. Composition of the participant groups varied according to the methodologic step applied. Whereas the regional maturity assessment process (see section 2.2) was led by the respective in-house dietician as regional expert offering relevant insights to nutritional processes and needs of their residents, participants for the step-based co-creation process (see section 2.4) comprised of stakeholders and experts, which were already involved within the overall project context and/or who played an important key role in this regard. All participants gave their informed consent to participate in the project in written form and approval was obtained from the local ethics committee (No. 34011 ex 21/22 1450-2021).

### 2.2. Regional maturity assessment

The regional maturity assessment was conducted during the kick-off process for each LTC location involved into the project independently, in order to evaluate regional capacities and barriers for driving the nutrition care pathway development. This first methodological step was performed in a needs-assessment approach [27–29]. Assessment was performed within focus-group settings to enable an effective self-critical reflection process in the course of an interactive format [30].

### 2.3. Matching with existing care pathways

An intermediate methodological step was conducted in the course of a matching process with relevant existing nutritional care pathways in the view to align structural and setting-related overlaps of processes and competencies. Analysis and mapping of care pathway sections was performed by three researchers (RRW, SLR, CH) and disagreements were solved by discussion until consensus was reached.

### 2.4. Step-based pathway co-creation with stakeholders

In pursuance of generating ideas and concepts through joint knowledge and experiences, a gradual co-creation process according to the meta-model of “joint space of creation” [31] was applied, also following the principle of design thinking for healthcare innovations [32]. In this regard, results from the maturity assessment (see section 2.2) as well as the matching process (see section 2.3) were grouped into a first synopsis according to care episode and respective professional competencies.

The subsequent co-creation process was threefold with an initial online consultation round in the project core group followed by a written consultation and concluded by an iterative, multi-stakeholder co-creation process within an online workshop. Chosen workshop format was focus-group design as focus is being laid on discussion of particular topics initiated by the project lead and generation of data, insights as well as exploration of research questions within group interaction [33]. Participants were divided into smaller sub-groups with an emphasized regional distribution according to nursing home locations in order to achieve highest possible practicability and context orientation of results as well as potential regional adaptations of the care pathway, if required. Results were incorporated into the synopsis by three researchers (RRW, GW, SLR) and reviewed as well as approved within a concluding supervision process, resulting in the final version of the nutritional care pathway.

### 2.5. Evaluation

Considering the aim and characteristics of the project activities, a qualitative evaluation design with semi-structured interviews was used, making use of different perspectives of stakeholders with personal experiences within the nutritional care context. Performance and implementation indicators were selected from the Integrated Care Performance Assessment Framework [34], which was developed on the basis of formerly validated frameworks.

### 2.6. Data collection, analysis and reflexivity procedures

Data collection was performed at different settings, with the regional maturity assessment (section 2.2) taking place at each long-term care facilities’ location and the pathway co-creation workshop (see section 2.4) as online event facilitated via the WebEx application. Evaluation interviews (see section 2.5) were conducted using a hybrid methodology, accommodating the preferences of each interview partner by offering the option of either face-to-face interviews at their respective workplaces or online interviews via WebEx. An open and flexible approach was applied for qualitative data gathering during the regional maturity assessment and pathway co-creation process: Researchers provided only a respective broad structural framework, with a thematic needs assessment structure to assess regional maturity for integrated nutritional care on the one hand and a conceptual sketch to guide subsequent pathway co-creation on the other hand. In contrast to that, interview questions were guided by a semi-structured interview guide following the principle of exploratory research to ensure consistency while allowing for in-depth exploration of participants’ perspectives. Audio recording was used for data collection and field notes were made during regional maturity assessments, the focus group session for pathway co-creation and all evaluation interviews by one researcher (SLR). Data analysis was conducted using summarizing content analysis [35,36] by employing MAXQDA software. Applied transcription system for received audio material was the smooth verbatim transcript, which represents original wording but filling utterances and decorating words are left out and dialects are translated into standard language. A four-step process model for summarizing content analysis was carried out by two researchers (SLR, CH) under the supervision of two further researchers (RRW, GW); starting with 1) determination of units of analysis, followed by 2) paraphrasing of content-bearing text passages, 3) generalization and reduction of paraphrases, and final 4) configuration and re-testing of statements as a category system [35,36]. Against this background, every complete statement by any focus group participant referring to in-house practices of nutritional care was determined as unit of analysis in terms of pathway development and every complete statement by an interviewed stakeholder referring to one or several of selected evaluation indicators was determined as unit of analysis regarding study evaluation. Abstraction levels were systematically determined through an iterative process of data engagement and categorization, moving from explicit expressions from participants as individual cases towards overarching themes and conceptual patterns, ensuring that statements remain grounded in the original data while increasing conceptual depth for the analysis. Reduction was realized by eliminating double or insignificant statements and combining similar or supplementary statements. Category re-testing was performed by systematically re-assessing them against the original material to ensure their consistency and validity.

Various reflexivity procedures were applied to secure methodological rigor. The project core team was aware that their professional background in geriatrics, nutritional care and integrated care may shape their understanding and interpretation of research findings and practices [37]. Therefore, two reflexivity meetings were held to allow for discussions and reflection of assumptions during the whole process, one preparatory meeting before kick-off to discover potential biases, and one reflective meeting during pathway revision process. Moreover, project participants were given the opportunity to reflect upon the whole project and their role in it by evaluating their personal experience with the initiative. Lastly, a final project meeting gave space for concluding reflections on project outcomes, the overall research process and if initial expectations were met.

## 3. Results

### 3.1. Results from the regional maturity assessment

Three focus group sessions were held from July to August 2021 with 29 participants in total (of 39 invited, which corresponds to a participation rate of 74%) and a duration between 2.5 hours and 4 hours. A total of 263 content-bearing text passages were paraphrased and the subsequent generalization and reduction process led to a total of 22 significant paraphrases generated. The extrapolated segments were systematically allocated and categorized according to following respective care episodes in the first draft of the nutritional care pathway: Screening, (Dietetic) Assessment, Diagnostics, Treatment Goal Definition, Intervention, Monitoring/Evaluation.

### 3.2. Results of the matching process with existing nutritional care pathways

One existing care pathway (n = 1) was identified relevant for the matching process, relating to nutritional care of geriatric patients in the primary care setting [38]. Depicted algorithms of action encompassing inter-professional collaborative practice in nutritional care, which are provided by the general practitioner (GP) in combination with dieticians and/or other relevant professions [38] and describing care procedures with relevance for the long-term care setting, were taken into account for the planned nutritional care pathway.

### 3.3. Results of the step-based pathway co-creation with stakeholders

Results from previous methodological steps were incorporated into a first synopsis and a first consultation round was conducted within an online meeting of the project core group (n = 6: RRW, GW, SLR, JF, CH, LP) followed by a written review of the extended core group (n = 8: RRW, GW, SLR, JF, IW, ED, CH, LP). Based on the material provided, action processes were structured and prepared for the next consultation round. The third consultation round within an online workshop was attended by 15 participants divided into three sub-groups. Qualitative data analysis [36] resulted in 88 paraphrases generated from content-bearing text passages. Generalization and reduction led to 56 final responses to be considered for pathway refinement, structured in 7 categories and 12 sub-categories. The revised synopsis of the pathway was reviewed within an internal supervision (n = 6: RRW, GW, SLR, BW, KPS, BB) and approved as final version, now structured into seven care episodes: Admission & Anamnesis, Screening, Nutritional Assessment, Nutritional Medical Intervention, Monitoring & Evaluation, End-of-Life Nutritional Care. The final inter-professional nutritional care pathway can be retrieved from the Supporting Information files (S2 Pathway).

### 3.4. Evaluation results and outlook on nutritional care pathway implementation

Four evidence-based indicators were chosen for nutritional care pathway evaluation: “Staff experience of the integrated care initiative”, “acceptability”, “appropriateness” and “feasibility” [34]. Additional indicators concerning changes in collaboration/communication, as well as success factors and/or barriers were added. A total of 12 persons was interviewed, divided into the following professional groups: dietetics (n = 3), nursing care/nursing management (n = 6), medical and care management (n = 2) and operational management (n = 1). Length of interviews ranged from 6 minutes to 33 minutes with a mean duration of 16 minutes.

Qualitative content analysis [35,36] led to a total of 342 paraphrases generated. Within subsequent generalization and reduction processes, a final 116 significant statements were retrieved and structured into an indicator-led category system consisting of the indicators mentioned above.

Regarding the indicator of “staff experience of the integrated care initiative”, results showed a promising effect independent from initial doubts raised by participants at kick-off of the care pathway development process. The initiative was regarded a successful basis, paving the way for further development and helping to overcome identified weaknesses.

In terms of “acceptability”, the developed care pathway was considered as an adaptive basic framework and helpful tool, providing guidance and evidence for an eased nutritional practice. Although the nutritional care pathway may contribute to quality increase, its success mainly depends on acting persons on-site and practice implementation of the care pathway still remains a challenge.

In relation to “appropriateness”, the methodological approach towards development of the nutritional care pathway was regarded informative and inclusive. Although local anchoring of the tool may be facilitated by fixed structures within the settings and the applied bottom-up approach promotes continuous development, further sustainable anchoring of the tool is desired with help of external support. The impact of the implemented integrated care initiative still depends on direct involved and future acting persons and more projects as well as initiatives were regarded necessary to increasingly raise awareness of the topic.

Evaluation results revealed more critical perspectives with regards to the “feasibility” indicator. Although the output of the nutritional care pathway enables to gain experiences in integrated nutritional care and facilitates necessary adaptations in daily care, realization in practice remains a serious factor. Moreover, documentation and evaluation possibilities are required for a successful long-term practice implementation. The existing system barrier and preservation of motivation may also interfere with feasibility of the interprofessional nutritional care pathway. Altogether, a sustainable practice realization, adaptation and evaluation as well as further thematic support beyond project end remain critical factors.

However, evaluation results within the indicator “changes of collaboration and communication processes” showed encouraging effects. The topic of nutritional care was regarded as dialogue, discussion and chance that is now treated more interdisciplinary and increased its presence in daily communication of the care teams. Besides positive experiences within the initiative that boosted solidarity, communication has evolved and is now characterized by a more participative and professional nature. The initiative allowed for an adoption of good practices across all locations and the nutritional care pathway serves as an important tool for cross-location integration. Nevertheless, some regional challenges are not solved yet with some further handling of challenges remaining unclear and the expected implementation success of the integration initiative still remains open and requires further evaluation.

Highlighted success factors of the initiative were its inclusive approach, a previously high awareness of the topic and preceding good collaboration as basis, as well as flexibility, impulses for change and an appreciative social interaction. Mentioned barriers comprised the COVID-19 pandemic that impaired project conduct, limited resources, the perceived setting-specific system barrier, the serious economic situation, high documentation efforts as well as long formal processes of quality assurance. The topic of integrated nutritional care in long-term care was overall regarded as being very challenging and doubts about problem solving via the presented initiative still remain.

Supplementary S1 Table in the appendix presents a more in-depth overview of the evaluation results.

## 4. Discussion

The current paper describes the development and implementation process of a contextualized and inter-professional nutritional care pathway in LTC facilities in Austria, grounded in a distinct implementation science framework [22]. Nursing home residents typically represent a heterogeneous population with divergent care and nutritional needs, necessitating an inter-professional approach that bundles complementary perspectives, skills and care processes [39,40]. The pathway aims at guiding the care team around the residents to choose needs-oriented and evidence-based interventions based on a person-centered nutritional care approach.

As care pathways are regarded as complex interventions being designed with the intent to function within a complex system, understanding the care context in which care is provided is an important prerequisite for effective and sustainable care pathway implementation [26]. The European Pathway Association (EPA) provides a framework that guides care pathway development and application in relation to care context, amongst others [26]. As part of this study, analysis of the respective care contexts was conducted within a preceding maturity assessment, depicting strengths, weaknesses, opportunities and threats of nutritional care provision across all three nursing home locations, taking into account inner and outer organizational factors that shape the operational context [41,42] and outline feasibility of pathway implementation. Interestingly, our data demonstrated a huge variety in context related care ecosystems already within three LTCs involved, although all three units are run under the umbrella of one trust. This observation, once more, underlines the importance and value of the first step also recommended by the European Pathway Association (EPA) framework [26].

In addition to this, the implementation strategies from the Expert Recommendations for Implementing Change (ERIC) project [22] provided further valuable methodological guidance for tailoring and executing the nutritional care pathway development process. Several strategies were particularly instrumental in supporting a contextual and flexible approach. First, the strategy to “assess for readiness and identify barriers and facilitators” [22] – consistent with the first step of the EPA framework [26] - was applied through the initial maturity assessment, allowing for a structured understanding of contextual enablers and challenges across the three LTC facilities. Second, the strategy to “capture and share local knowledge” [22] was realized within focus group sessions where key participants shared local knowledge on how to effectively execute nutritional care in everyday practice in their specific setting. Third, “consensus discussions” [22] were held within the co-creation process of the care pathway development, ensuring appropriate design of the innovative care pathway. Fourth, executive boards [22] have been involved throughout the whole process, with the management board of the Styrian hospital trust KAGes m.b.H. acting as governing structure and being in charge of final approval of the results. Fifth, the strategy of “facilitation” [22] was applied by engaging in interactive solution finding based on the needs recognized in the initial maturity assessment. Finally, recognizing the dynamic nature of implementation, the “promote adaptability” [22] strategy was key in allowing for iterative adjustments based on ongoing feedback and contextual needs. Employing the ERIC framework [22] helped to develop an individual multicomponent approach towards integrated nutritional care implementation, as the discrete strategies presented within the compilation serve as solid methodological pillars for implementation science.

In general, the nature of integrated care is characterized by its diversity of methods, models and processes within and across different care settings. This directly impacts assessment and quality assurance frameworks related to integrated care processes [43,44]. The challenge of evaluating and measuring the impact of integrated care initiatives and interprofessional collaborative practice has been thoroughly discussed in literature [45–48], both for hospital as well as nursing home care processes [40]. From a conceptual perspective [49], nutritional care delivery and its quality needs to be evaluated from the side of structural and outcome indicators [40]. The study presented in this publication focused on integration of nutritional care delivery in the LTC facilities involved in the project within implementation of the innovative integrated care pathway shown in the Supportive Information files of this paper. Generally speaking, evaluation results reveal a positive impression of the integrated nutritional care initiative. However, one have to bear in mind that the results reflect perspectives from different professional silos that are characterized by pre-existing opinions and experiences. Moreover, working routines that are embedded in pre-established structures or frameworks and may be difficult to adapt or change further pose a factor that needs to be considered in terms of care integration initiatives. This is also reflected in the literature, as engagement of staff is regarded crucial when designing nutritional interventions in healthcare settings [50]. Corresponding to this, evaluation results reveal that long-term implementation into practice routine is regarded a major challenge. Still, valuable lessons learned emerged that may facilitate scaling-up aspirations for integrated care initiatives. One major condition for success is to maintain a flexible approach that fits the contextualized integration strategy and allows for adaptations and corrections along the road. In this sense, a continuous improvement process is made possible that ensures achievements and avoids losing track within a too rigid procedure. Moreover, as previous integrated care implementation experiences confirm, more clearly defined and smaller changes exhibit a higher probability of successful implementation [51].

As implementing changes in health and social care organizations encompass planned as well as unplanned measures [51], another essential scaling-up recommendation is to early engage in a method of barrier or challenge identification [52]. Within such assessment, a deeper understanding of potential impacts and influences will be gained and integrated care progress may be secured more easily.

Implementing and scaling-up integrated care efforts does not require to “reinvent the wheel”, in fact it is about finding, planning and realizing an individual and contextualized approach within the guidance of prevailing frameworks. The presented initiative has been deployed using the developed methodology from the EU-funded VIGOUR project (Grant Agreement Number 826640) [53], however, interested parties will find several different guiding frameworks that comprise the building blocks for integrated care development in the literature, such as the Rainbow Model of Integrated Care [54], the (expanded) Chronic Care Model [55] or WHO’s Innovative Care for Chronic Conditions Framework [56], amongst others. The purpose of these frameworks is to navigate through the integrated care system and simultaneously allowing room for contextualized practical implementation addressing emerging care needs.

A significant context orientation lies in the nature of implementation science, as research not only takes into account individual patients or subjects, but has to carefully analyze settings, facilities, organizations, communities and even the macro level of policy environment or health care systems as well [57,58]. In this regard, another prerequisite for successful implementation research is the high degree of participation of individuals and integration of structural conditions. As implementation research targets innovation and intervention at structural level where key stakeholders are operating as local experts, their participation is essential for successful implementation [59].

The present multi-method pathway development process displays strengths and limitations.

The implementation of integrated care measures, such as the inter-professional nutritional care pathway described in this paper, requires procedures, structures and mechanisms that are tailor-made, addressing contextualized issues and circumstances [60]. The inter-professional nutritional care pathway presented here, is a custom-made tool for integrated nutritional care of LTC residents, approaching issues and routines explicitly for the three LTC facilities of the Styrian hospital trust KAGes.

By applying a concept of co-creation including all stakeholders in health and social care, shared commitments towards improvement of nutritional care in long-term care are made possible. Co-creation in health and social care serves as a promising methodological approach enabling cross-fertilization among stakeholders and shedding light on perspectives and attitudes transcending individual and isolated frames of references [61]. The methodological approach presented is grounded in implementation science, utilizing the ERIC taxonomy [22] to achieve sustainable and contextually meaningful outcomes.

However, although an iterative, multi-stakeholder design process was applied, patients’ perspectives are still lacking. There is growing evidence that patient and/or consumer participation are relevant for dealing with current challenges in health and social care provision and enables them to actively contribute on their individual care journey [62,63]. Valuable patient involvement shows the potential to increase and reinforce project outcomes in health care research, as patients are equipped with unique expertise when it comes to their own health and individual experiences in their healthcare journey [64]. Furthermore, patient or service user involvement and engagement helps to bridge research and practice, to identify issues and enables a greater needs orientation and co-production of care services [65,66]. One essential prerequisite in this regard is a mutual understanding of the concept and dimensions of patient-centered care provision [67]. There are several guidelines and recommendations addressing policy and researchers alike to foster inclusion, empowerment and participation of patients [64,65]. In this sense, important key factors are for example the right to involvement, resources and capacity building [64]. Burgher et al. presented more specific recommendations for public involvement in care home research. As a result, a successful approach towards patient and public involvement (PPI) comprises of five main subjects: 1) appreciating stakeholders’ perspectives; 2) being aware of the multi-dimensional research context; 3) secure inclusiveness and transparency; 4) stay flexible and able to adapt; 5) make use of resources and extended support [68].

Finally, evaluation results of care pathway implementation, giving insight into outcomes, effects, sustainability and necessary adaptations of the intervention [69], are still pending. Further evaluation research is needed in this regard in order to present comprehensive long-term results underpinning the method applied. Furthermore, deeper understanding about the dynamic nature and impact of this context-specific innovation will be generated, enriching the current evidence base within the field of implementation science [57].

## 5. Conclusion

The present study gives insight into a collaborative approach to develop and implement an inter-professional, evidence-based nutritional care pathway, bridging health and social care around the complex needs of older residents in three LTC facilities across Styria, Austria. Early evaluation results show promising effects, however, further in-depth evaluation is required to consolidate and eventually adapt integrated nutritional care provision in the context of a long-term quality assurance approach.

## Supporting information

S1 Table Overview on evaluation results. (PDF)

S2 Pathway Interprofessional nutrition care pathway. (PDF)

S3 Checklist SQUIRE 2.0: Checklist. (PDF)
