# Supplementary material for: Integrated nutritional care in long-term care: From theory to evidence-based practice
Source: PLoS One. 2025 May 30;20(5):e0323596. doi: 10.1371/journal.pone.0323596 (PMC12124848; doi:10.1371/journal.pone.0323596)
Supplement: S1 Table — (PDF) [file pone.0323596.s001.pdf]

Table S1 Overview on Evaluation Results

Indicator	***Evaluation Results***
**Staff experience of the integrated care initiative**	- Knowledge transfer as appreciative experience and sensitization<br>- Confirmation of further development<br>- Overcoming of identified weaknesses<br>- Development of a successful basis<br>- Required significant cooperation (resources)<br>- Uncertainties about results, direction, goals at the beginning<br>- Doubts concerning resources expenditure and risk of insufficient participation at the beginning
**Acceptability**	- Nutrition care pathway as adaptive basic framework and helpful tool for quality residential care, quality increase and securing competitive advantage<br>- Nutrition care pathway as guideline for an eased practice and as evidence, e.g. nursing home reviews<br>- Nutrition care pathway as improved filter tool, as current screening requires too many resources<br>- Challenge of practice implementation of the nutritional care pathway<br>- Nutrition care pathway confirms routines in practice<br>- Success depends on acting persons on-site
**Appropriateness**	- Informative and inclusive methodological approach<br>- Local anchoring via fixed structures and bottom-up approach<br>- Sustainable anchoring via external support desired<br>- Impact often remains with direct involved and future acting persons → puzzle piece from institutional-wide perspective<br>- Necessity of more projects and initiatives to raise awareness<br>- Initiation of a continuous development
**Feasibility**	- Good output for practice<br>- Practice realization as critical factor: Uncertainty at the beginning<br>- Practice realization enables gain of experiences and adaptations<br>- Practice realization requires documentation and evaluation possibilities<br>- System barrier requires detailed agreements for practice realization

	- Motivation and practice realization as challenges beyond project end<br>- Critical factors: Sustainable practice realization, adaptation and evaluation as well as further thematic support beyond project end
**Changes of collaboration and communication processes**	- More intense inter-disciplinary treatment of the topic<br>- More presence of topic in nursing care communication<br>- Nutritional care as dialogue, discussion and chance<br>- Dialogue facilitation and impulse for reflection as collaboration support<br>- More detailed analysis of processes and procedures<br>- Improved participative, professional communication (also with management level), basis for discussion, practical orientation and raised awareness<br>- Exchange, reflection and evaluation of previous practices<br>- Collaboration of dietetics and Adoption of good-practices across locations<br>- Inter-professional division of work desired<br>- Confirmed awareness of underrepresentation of the topic<br>- Positive solidarity experiences<br>- Good team collaboration and communication in advance (dependent from location between dietetics and care or between dietetics, care and GPs, respectively)<br>- Regional situation not solved yet, further handling of the situation not clear yet<br>- Nutritional care pathway as important tool for integration<br>- Expected success of integration not clear yet
**Success factors and/or barriers**	<u>Success factors:</u><br>- Top-down prioritisation by management board<br>- Successful basis via previously well collaboration<br>- Development of a red thread<br>- Enthusiasm and initiative of project lead<br>- Great engagement and interest of involved persons<br>- Competency, exchange of information and opinions, communication<br>- Consideration of all participants<br>- Previously high awareness of topic<br>- Appreciative social interaction, common dialogue for sensitization

	- Impulse for change and persuasive efforts<br>- Inter-professional good collaboration<br>- Flexibility<br><br><u>Barriers:</u><br>- COVID-19 Pandemic<br>- Limited resources<br>- Existing system barrier between hospital and nursing home (additional effort in nursing home)<br>- Shared kitchen with hospital and economic aspects as barriers for individual nutritional care<br>- Economic situation<br>- Taboo subject aging and death (for relatives)<br>- High documentation efforts<br>- Long formal processes of quality assurance<br>- Challenging topic<br>- Doubts about the possibility of problem solution via project
