# Supplementary figures and images for: Integrated nutritional care in long-term care: From theory to evidence-based practice
Source: PLoS One. 2025 May 30;20(5):e0323596. doi: 10.1371/journal.pone.0323596 (PMC12124848; doi:10.1371/journal.pone.0323596)

# Interprofessional Nutrition Care Pathway (I)

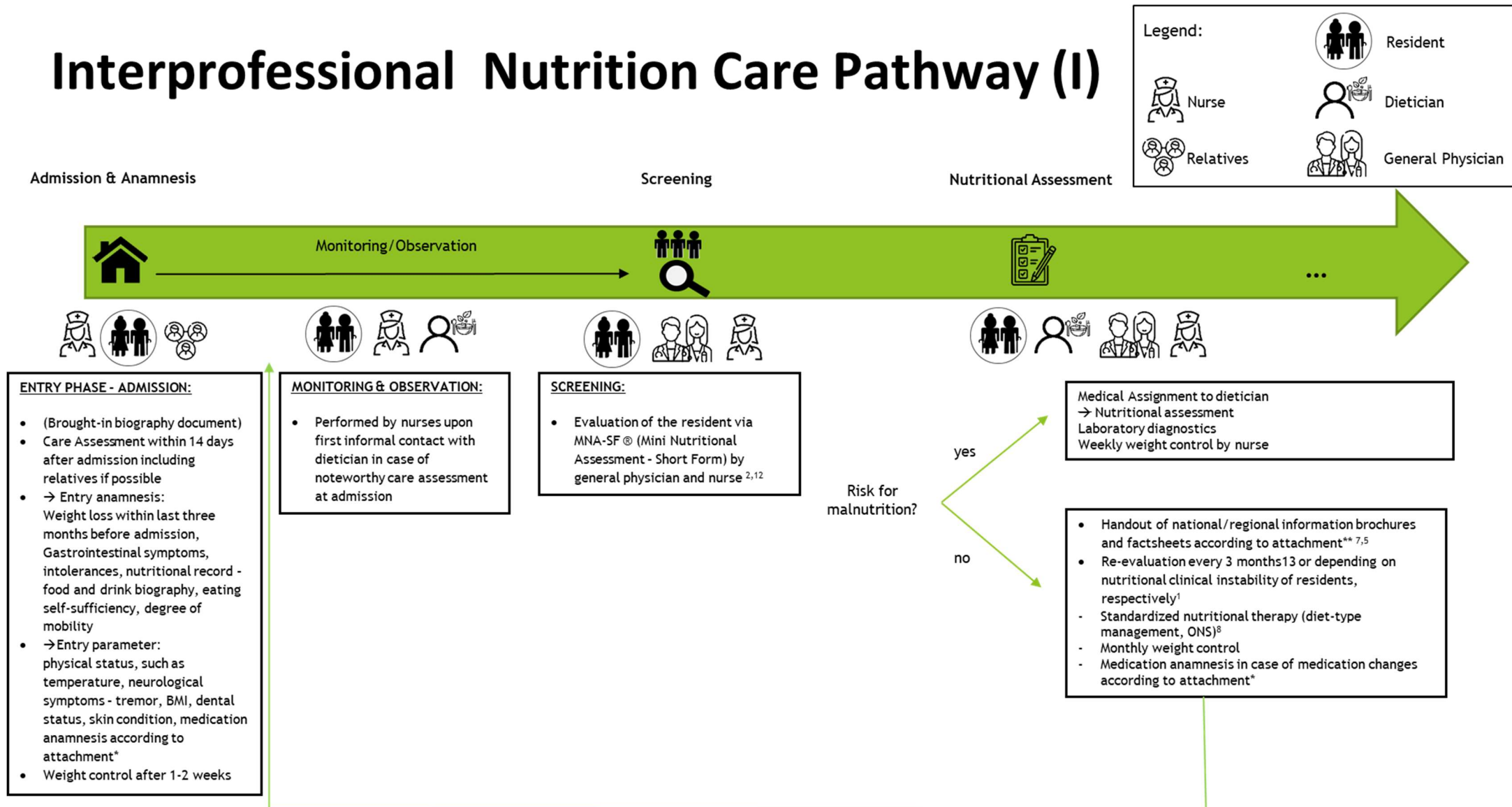

# Interprofessional Nutrition Care Pathway (II)

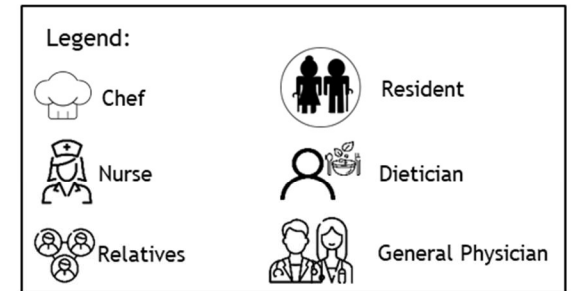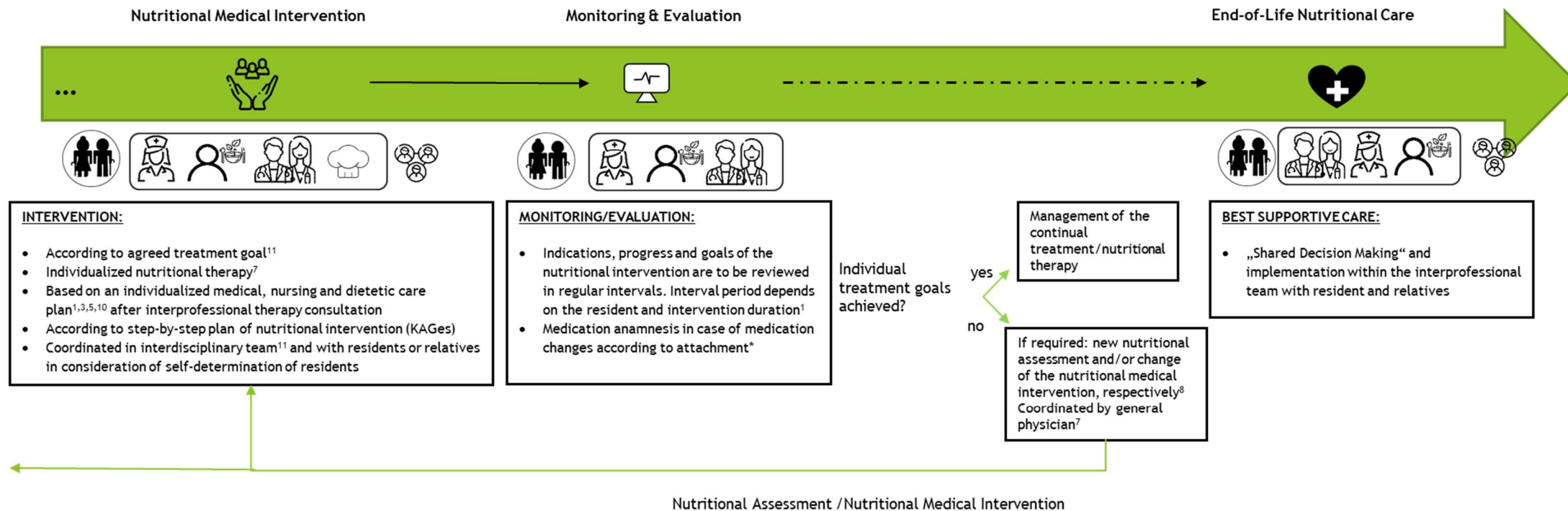

Supplement: S2 Pathway — (PDF) [file pone.0323596.s002.pdf]
